# A randomized Phase II trial evaluating efficacy, safety, and tolerability of oral BI 409306 in attenuated psychosis syndrome: Design and rationale

**DOI:** 10.1111/eip.13083

**Published:** 2020-12-22

**Authors:** Richard S. E. Keefe, Scott W. Woods, Tyrone D. Cannon, Stephan Ruhrmann, Daniel H. Mathalon, Philip McGuire, Holger Rosenbrock, Kristen Daniels, Daniel Cotton, Dooti Roy, Stephane Pollentier, Michael Sand

**Affiliations:** ^1^ Department of Psychiatry and Behavioral Sciences Duke University Durham North Carolina USA; ^2^ VeraSci Durham NC USA; ^3^ Department of Psychiatry Yale University New Haven Connecticut USA; ^4^ Department of Psychology Yale University New Haven Connecticut USA; ^5^ Department of Psychiatry and Psychotherapy University of Cologne Cologne Germany; ^6^ Department of Psychology UCSF School of Medicine San Francisco California USA; ^7^ Department of Psychosis Studies, Institute of Psychiatry, Psychology and Neuroscience King's College London London UK; ^8^ Boehringer Ingelheim Pharma GmbH & Co KG Biberach an der Riss Germany; ^9^ Boehringer Ingelheim Pharmaceuticals Inc. Ridgefield Connecticut USA; ^10^ Boehringer Ingelheim International GmbH Ingelheim Germany

**Keywords:** cognition, early intervention, phosphodiesterase, psychotic disorders, schizophrenia

## Abstract

**Aim:**

Attenuated psychosis syndrome (APS), a condition for further study in the Diagnostic and Statistical Manual of Mental Disorders‐5, comprises psychotic symptoms that are qualitatively similar to those observed in schizophrenia but are less severe. Patients with APS are at high risk of converting to first‐episode psychosis (FEP). As evidence for effective pharmacological interventions in APS is limited, novel treatments may provide symptomatic relief and delay/prevent psychotic conversion. This trial aims to investigate the efficacy, safety, and tolerability of BI 409306, a potent and selective phosphodiesterase‐9 inhibitor, versus placebo in APS. Novel biomarkers of psychosis are being investigated.

**Methods:**

In this Phase II, multinational, double‐blind, parallel‐group trial, randomized (1:1) patients will receive BI 409306 50 mg or placebo twice daily for 52 weeks. Patients (*n* = 300) will be enrolled to determine time to remission of APS, time to FEP, change in everyday functional capacity (Schizophrenia Cognition Rating Scale), and change from baseline in Brief Assessment of Cognition composite score and Positive and Negative Syndrome Scale scores. Potential biomarkers of psychosis under investigation include functional measures of brain activity and automated speech analyses. Safety is being assessed throughout.

**Conclusions:**

This trial will determine whether BI 409306 is superior to placebo in achieving sustainable remission of APS and improvements in cognition and functional capacity. These advances may provide evidence‐based treatment options for symptomatic relief in APS. Furthermore, the study will assess the effect of BI 409306 on psychotic conversion and explore the identification of patients at risk for conversion using novel biomarkers.

## 1 INTRODUCTION

International research has identified a subgroup of individuals who are in a clinical high‐risk state for psychosis. These individuals meet this diagnosis because they fulfil the criteria in the Diagnostic and Statistical Manual of Mental Disorders (DSM)‐5 for attenuated psychosis syndrome (APS), which has been listed as a condition for further study (American Psychiatric Association, 2013). Individuals who are in the clinical high‐risk state exhibit subclinical cognitive, behavioural, emotional, and motor impairments that are less severe than those experienced in psychosis (Calkins et al., 2014) and have a poorer quality of life than healthy controls (Fusar‐Poli et al., 2015). These individuals often receive psychiatric medications for their condition, as shown in the North American Prodromal Longitudinal (NAPLS) study, which demonstrated that 51% of patients who are at clinical high risk for psychosis received psychiatric medication at baseline (Walker et al., 2009). Similarly, a US‐based retrospective claims study looking at healthcare resource utilization in individuals <5 years before receiving a diagnosis of schizophrenia found that these individuals had more frequent encounters with healthcare providers and more claims for antipsychotics than healthy comparators (Wallace et al., 2018).

Individuals with APS are more likely to develop a psychotic disorder than the general population and of those in the clinical high‐risk state, 20 and 23% are likely to convert to psychosis at 2 and 3 years, respectively (Salazar de Pablo et al., 2019). However, a large proportion of patients who are in a clinical high‐risk state do not convert to psychosis (Addington et al., 2011). Addington et al., 2011 demonstrated that many (24%) appear to remit symptomatically and to function in the normal range, while others (20%) may remain symptomatic and/or functionally impaired (Addington et al., 2011). An additional 21% of patients were not assessed at follow‐up as they had received antipsychotic therapy during the study, which may have affected their symptoms (Addington et al., 2011). However, the effects of any antipsychotic may have been limited based on a previous study showing that the antipsychotic olanzapine did not significantly prevent conversion in patients at high risk of psychosis (McGlashan et al., 2006). Another study has also shown that a much greater proportion (72.4%) of clinical high‐risk patients who were nonconverters, remitted at follow‐up (median 53.7 months, range 13.9–123.7 months) (Michel et al., 2018). However, many of these exhibited functional problems (Michel et al., 2018) demonstrating the need for treatment options in this population.

Given that psychosis is harmful to patients and their families (Hastrup et al., 2013; Szkultecka‐Debek et al., 2016), the European Psychiatric Association (EPA) has recommended regular assessment of the mental state of high‐risk patients to allow for early detection and prompt intervention, with the goal of preventing first‐episode psychosis (FEP;Schmidt et al., 2015; Schultze‐Lutter et al., 2015). Similarly, in the United Kingdom, the National Institute for Health and Care Excellence (NICE) Guidelines recommend that all early intervention services engage people who are at clinical high risk, with the aim of alleviating presenting symptoms and reducing the risk of later psychosis (National Collaborating Centre for Mental Health, 2014). Overall, these recommendations indicate the need for treatment interventions that: (a) alleviate the cognitive, behavioural, and emotional symptoms that are present at the time of APS diagnosis and persist in a large group of patients, and (b) prevent conversion to psychosis.

As early detection of APS is recommended for the prevention of FEP (Schmidt et al., 2015; Schultze‐Lutter et al., 2015), improved prediction methods are needed to allow for early intervention (Addington et al., 2011). NAPLS have developed an individualized risk calculator to predict the probability of conversion to psychosis, based on symptoms, cognition, and social functioning at baseline (Cannon et al., 2016). Other potential predictors of psychosis include mismatch negativity and the P300 (P3a and P3b), two event‐related potential components shown to be reduced in patients with APS, particularly those who convert to full‐blown psychosis (Bodatsch et al., 2015; van Tricht et al., 2010). High‐risk patients also exhibit abnormal speech production, which has been associated with reduced glutamate levels within the hippocampus versus controls (Stone et al., 2010). Additional biomarkers of APS include inflammatory cytokines and increases in cortisol (Perkins et al., 2015). Overall, as early detection has become a major goal in the field of psychiatry, novel biomarkers of APS and prediction methods such as machine learning (Mechelli et al., 2017), multimodal prediction (Clark et al., 2016; Schmidt et al., 2017) and dynamic prediction (Yuen et al., 2019) are needed.

At present, there is limited evidence for pharmacological interventions in APS or for the prevention of FEP (Davies et al., 2018). Although the mechanisms underlying the onset of psychosis are unclear, there is evidence that synaptic pruning (removal of weak synapses), a process that occurs during postnatal and adolescent brain development, may be involved in the onset of psychosis (Stoneham et al., 2010). In particular, deficiencies in *N*‐methyl‐d‐aspartate (NMDA) receptor‐dependent mechanisms of neuroplasticity, such as long‐term potentiation (LTP), may result in an overabundance of weak synapses that have not been sufficiently strengthened by experience, resulting in excessive synaptic pruning during adolescence (Mathalon, 2014). Excessive synaptic pruning due to deficient NMDA receptor‐dependent synaptic plasticity has been considered a possible mechanism underlying the accelerated loss of cortical grey matter observed in clinical high‐risk patients during their transition to psychosis (Cannon et al., 2015).

**FIGURE 1:**
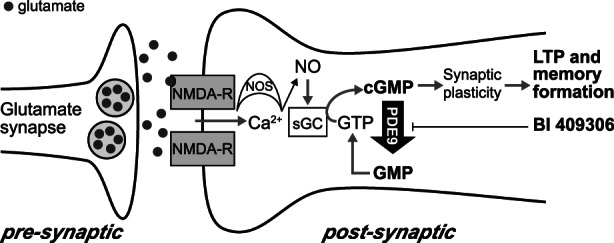
Mode of action for PDE9 and BI 409306. Ca^2+^, calcium; cGMP, cyclic guanosine 3′,5′‐monophosphate; GTP, guanosine triphosphate; LTP, long‐term potentiation; NMDA‐R, *N*‐methyl‐D‐aspartate receptor; NO, nitric oxide; NOS, nitric oxide synthase; PDE9, phosphodiesterase‐9; sGC, soluble guanylate cyclase. Figure adapted from reference Moschetti et al. (2016) Copyright © 2016 Boehringer Ingelheim. British Journal of Clinical Pharmacology published by John Wiley & Sons Ltd on behalf of British Pharmacological Society. This is an open access article under the terms of the Creative Commons Attribution‐Non‐Commercial License

BI 409306 is a potent and selective phosphodiesterase‐9 (PDE9) inhibitor that has been shown in preclinical studies to strengthen synaptic plasticity by promoting hippocampal LTP (Figure 1) (Dorner‐Ciossek et al., 2015). BI 409306 is well tolerated in healthy volunteers and patients with schizophrenia (Boland et al., 2017; Brown et al., 2015; Moschetti et al., 2016; Wunderlich et al., 2015). Here we describe the design of an adequately powered, industry‐sponsored, proof‐of‐concept trial to investigate the efficacy, safety, and tolerability of BI 409306 versus placebo in patients with APS. The study is designed to show superiority of BI 409306 over placebo in achieving remission of APS and improvement in cognition and functional capacity. The assessment of exploratory biomarkers of psychosis, including automated speech analysis and an electroencephalogram (EEG) substudy will be described. In addition, following previous BI 409306 Phase I and II trials showing that eye disorders were frequently reported as transient, mild to moderate adverse events (Brown et al., 2018; Moschetti et al., 2015; Moschetti et al., 2018; Wunderlich et al., 2015), an ocular substudy is being conducted to further characterize the ocular safety of BI 409306. If successful, the trial may provide the following two advances to the field: a new treatment option for patients with APS, and new biomarkers to predict the onset of psychosis, which, in combination, will improve the identification, intervention, and prevention of FEP.

## 2 METHODS

### 2.1 Trial design

**FIGURE 2:**
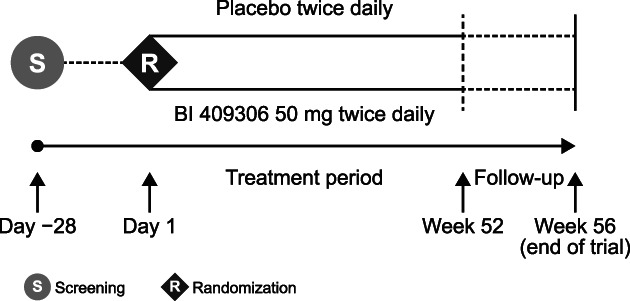
Overview of trial design

This Phase II, multinational, multicentre, randomized, double‐blind, placebo‐controlled, parallel‐group trial (BI trial: 1289‐0032, Clinicaltrials.gov: NCT03230097) was initiated in September 2017 and recruitment is ongoing. Three hundred patients with APS are planned to be enrolled from ~50 trial centres in Canada, the United Kingdom, China, and the United States. Patients are randomized (1:1) in a blinded fashion using interactive response technology (IRT) at baseline to receive either BI 409306 50 mg or placebo twice daily, for 52 weeks, with a 4‐week follow‐up (Figure 2). Randomization will be stratified by baseline use of antipsychotics, not by study site. The trial consists of 24 visits: Visit 1 (screening, Week −4), Visit 2 (baseline, Week 0), Visits 3–8 (Weeks 1–6, every week), Visits 9–23 (Weeks 9–52, every 3 weeks), and follow‐up (Week 56). Every other visit consists of a phone visit to minimize patient burden. If worsening of symptoms is suspected, an unscheduled in‐clinic visit is arranged. At the baseline visit, medication assignment will be provided through IRT. Patients, investigators, and everyone involved in trial conduct or analysis will remain blinded to the randomized treatment assignments until after database lock.

Treatment compliance will be measured by the scheduling of interim phone visits, tablet counts, and the utilization of a medication adherence monitoring platform. This platform will use artificial intelligence on a smartphone to confirm study medication ingestion. In addition, built‐in reminders and a communication system will allow real‐time intervention in case of drug interruptions. Patients will follow a series of prescribed steps in front of the front facing smartphone camera to visually confirm ingestion of the medication. The amount of guidance that the device will provide to the patient will be automatically reduced as the patient becomes more proficient at using the application.

All patients are permitted to remain on their concomitant psychotropic medications and will receive a new or increased dose of antipsychotic medication if the investigator judges that the patient has experienced the onset of psychotic symptoms during the trial. Restrictions to concomitant medications include St. John's Wort, medications that may be altered by or interfere with BI 409306 administration, and medications that are strong–moderate Cytochrome P450 (CYP)1A2 inhibitors unless patients are genotyped as not being a poor metabolizer of CYP2C19.

If patients transition to FEP, they can continue to participate in the trial and stay on trial medication at the discretion of the investigator. Patients withdrawing from the study are asked to consent to be monitored for the occurrence of further episodes for the remainder of the trial. The trial conduct is in accordance with the ethical principles of the Declaration of Helsinki (World Medical Association, 2013), International Council for Harmonization of Technical Requirements for Pharmaceuticals for Human Use Good Clinical Practice guidelines (International Conference on Harmonisation of Technical Requirements for Registration of Pharmaceuticals for Human Use, 1996), and applicable country‐specific regulatory requirements. All patients will provide written, informed consent before undergoing study procedures.

### 2.2 Patients

Patients are 16–30 years of age (inclusive) and meet the diagnostic criteria for APS, as defined in the DSM‐5 (American Psychiatric Association, 2013) and determined by the Structured Interview for Psychosis‐Risk Syndromes (SIPS [McGlashan et al., 2010] version 5.6.1; dated January 5, 2017) at screening and diagnosis. Furthermore, SIPS is repeated at the final visit, whether at Week 52 or end of treatment, collect a more comprehensive evaluation of the patient at the final visit. All SIPS interviews are video recorded and if the rater concludes that the patient meets the diagnostic criteria for APS, both SIPS response form and video‐recorded interview will be reviewed by VeraSci (Durham, NC) to confirm APS diagnosis. Full eligibility criteria are described in Table A1, Supporting Information. All participants are receiving brief supportive psychoeducation (as standard of care) for the duration of the trial. Eligible patients are assessed using the NAPLS risk calculator, which is a web‐based tool used to predict the risk of psychotic conversion within 12 months and that can be regarded as a composite assessment of baseline severity. The NAPLS risk calculator produces a score of 0–1, corresponding to 0–100% predicted probability of transitioning to psychosis (Cannon et al., 2016). The assessment is based on age, Brief Assessment of Cognition in Schizophrenia (BACS): Symbol Coding, Hopkins Verbal Learning Test‐Revised, SIPS Items P1 and P2 (unusual thought content and suspiciousness), and Global Functioning: Social scale scores.

### 2.3 Assessments and endpoints

All clinical and cognitive assessments will be completed by raters with significant experience with psychotic populations and who are trained and certified on the assessments by experts who are employees or consultants to VeraSci. The rating scales and assessments used to assess symptoms, functional capacity, cognitive function, and safety in APS are described in Table 1. The primary endpoint is time to remission from APS within 52 weeks. Remission is defined by the reduction of all attenuated positive symptoms to levels that no longer support the diagnosis of APS (scores <3 on the P1 − P5 positive symptom items of the Scale of Prodromal Symptoms rating scale [SOPS]), maintained until the end of treatment, and in the absence of any other emerging psychiatric diagnosis. It is possible that some patients may show various symptom trajectories such as remission followed by brief relapse followed by remission. We will investigate these trajectories post hoc in exploratory analyses. Since there are various possibilities, it is likely that the number of participants following any single complex pattern will be small. Six weeks is the shortest period of remission possible, based on the interval between the penultimate and final symptom assessment. Safety is being assessed throughout the trial by an external data monitoring committee (DMC) and internally through regular safety updates and data‐quality reviews. Further details on the primary, secondary, and further endpoints are included in Table 2.

**TABLE 1:** An overview of the rating scales and assessments included in this trial

Name of rating scale or assessment	Measurement	Schedule
*Symptoms and functional capacity*		
CDS	Items of depression, hopelessness, self‐depreciation, guilty ideas of reference, pathological guilt, morning depression, early wakening, suicide, and interviewer's observed depression	Every 6 weeks
CGI‐I	Overall improvement compared with baseline from the physician perspective	Every 12 weeks
CGI‐S	Overall change in severity compared with baseline from the physician perspective	Every 12 weeks
C‐SSRS	Suicidal behaviour and suicidal ideation	Every visit
EQ‐5D‐5L	Current health status	At 52 weeks
GF: Social	Decline in social functioning in the past year	At 52 weeks
MINI	Diagnosis of psychotic and nonpsychotic DSM‐5 disorders	At 52 weeks
NAPLS risk calculator	Risk of psychosis	At 52 weeks
PANSS (Kay et al., 1987)	Severity of psychotic symptoms and disease progression	At 24 and 52 weeks
PGI‐I	Overall change in status compared with baseline from the patient's perspective	Every 12 weeks
SCoRS (Keefe et al., 2015)	Interview‐based measure of cognitive deficits and the degree to which they affect day‐to‐day functions	Every 12 weeks
SIPS (McGlashan et al., 2010)	Diagnosis of clinical high‐risk syndrome for psychosis and first‐episode psychosis	At screening, diagnosis and at the final visit (Week 52 or end of treatment)
SOPS (Miller, McGlashan, et al., 2003)	Severity and change of prodromal Positive, Negative, Disorganization and General symptoms. Positive items are used to diagnose APS, define remission and conversion to psychosis	Fortnightly until Week 6, every 6 weeks thereafter
Neurocognition		
BAC: SC	Speed of processing	Every 12 weeks
HVLT‐R	Recall of words by a patient from a list read aloud by the investigator	Every 12 weeks
Tablet‐based BAC (Atkins et al., 2017)	Neurocognition evaluation consisting of six tests (Verbal Memory, Digit Sequencing, Token Motor Task, Semantic and Letter Fluency, Symbol Coding, and Tower of London)	Weeks 18, 30, and 52
*Safety*		
Safety assessments	AE reporting, physical examinations, vital signs (blood pressure and heart rate), laboratory parameters, 12‐lead electrocardiogram, assessment of suicidality (C‐SSRS) and extrapyramidal symptoms (AIMS)	Various stages throughout study

Abbreviations: AE, adverse event; AIMS, Abnormal Involuntary Movement Scale; APS, attenuated psychosis syndrome; BAC, Brief Assessment of Cognition; CDS, Calgary Depression Scale; CGI‐I, Clinical Global Impressions of Improvement; CGI‐S, Clinical Global Impressions of Severity; C‐SSRS, Columbia Suicide Severity Rating Scale; DSM‐5, Diagnostic and Statistical Manual of Mental Disorders 5; EQ‐5D‐5L, EuroQol‐5 Dimensions‐5 Levels; GF, Global functioning; HVLT‐R, Hopkins Verbal Learning Test‐Revised; MINI, Mini‐International Neuropsychiatric Interview; NAPLS, North American Prodromal Longitudinal Study; PANSS, Positive and Negative Syndrome Scale; PGI‐I, Patient Global Impressions‐Improvement; SC, Symbol Coding; SCoRS, Schizophrenia Cognition Rating Scale; SIPS, Structured Interview for Psychosis‐Risk Syndromes; SOPS, Scale of Prodromal Symptoms rating scale.

**TABLE 2:** List of endpoints and exploratory biomarkers

Primary endpoint
Time to remission from APS within a 52‐week timeframe (a score of <3 on the P1–P5 positive) Symptom items of the SOPS and maintained until the end of treatment
Secondary endpoints
Time to onset of FEP within a 52‐week timeframe, confirmed by a central rating committee FEP defined as meeting one or both of two sets of criteria: ≥1 of the following positive symptoms (SOPS criteria) in the psychotic range of 6: Unusual thought content/delusional ideas Suspiciousness/persecutory ideas Grandiosity Perceptual abnormalities/hallucinations Disorganized communication AND either a symptom is seriously disorganizing or dangerous OR they occur for ≥1 h per day at an average frequency of 4 days per week for 1 month OR a new prescription or an increase in dose of an ongoing antipsychotic medication Change from baseline in everyday functional capacity as measured by SCoRS (Keefe et al., 2015) total score at Weeks 24 and 52
Change from baseline in the tablet‐based BAC (Atkins et al., 2017) composite T score at Week 52
Change from baseline PANSS (Kay et al., 1987) positive items score, negative items score, and total score at Week 52
Further endpoints
Change from baseline in SOPS total and domain scores at Week 52
Change from baseline in NAPLS risk calculator score at Week 52 (Cannon et al., 2016)
Change from baseline in the tablet‐based BAC subtest scores at Week 52
Change from baseline in BACS symbol coding and HVLT‐R score at Weeks 24 and 52
Change from baseline in CDS total score
Type of psychotic disorder diagnosis assessed by the MINI
Type of nonpsychotic DSM‐5 disorders assessed by the MINI
Change from baseline in CGI‐S score at Week 52
CGI‐I score over 52 weeks of treatment
PGI‐I score over 52 weeks of treatment
Frequency of positive AIMS scores at any time post‐baseline by treatment and baseline use of antipsychotics
Exploratory biomarkers
Variants in genes relating to schizophrenia and NMDA receptor signalling (Sekar et al., 2016)
Salivary cortisol at baseline at baseline and each clinic visit^a^
Blood inflammatory cytokines, including IL‐6, IL‐1β, and TNF‐α^a^
Blood BDNF as an outcome‐related pharmacodynamic biomarker^a^
Automated speech analysis at baseline, Weeks 18, 42, and 52

Abbreviations: AIMS, Abnormal Involuntary Movement Scale; APS, attenuated psychosis syndrome; BAC, Brief Assessment of Cognition; BACS, Brief Assessment of Cognition in Schizophrenia; BDNF, blood brain‐derived neurotrophic factor; CDS, Calgary Depression Scale; CGI‐I, Clinical Global Impression of Improvement; CGI‐S, Clinical Global Impressions Scale Severity; DSM‐5, Diagnostic and Statistical Manual of Mental Disorders 5; FEP, first‐episode psychosis; HVLT‐R, Hopkins Verbal Learning Test‐Revised; IL, interleukin; MINI, Mini‐International Neuropsychiatric Interview; NAPLS, North American Prodromal Longitudinal Study; NMDA, *N*‐methyl‐D‐aspartate; PANSS, Positive and Negative Syndrome Scale; PGI‐I, Patient Global Impression of Improvement; SCoRS, Schizophrenia Cognition Rating Scale; SOPS, Scale of Prodromal Symptoms; TNF, tumour necrosis factor.

^a^ Blood and saliva samples were collected at the following clinic visits: Visit 2 (baseline, Week 0), 10 (Week 12), 14 (Week 24), and end of trial (Week 52).

### 2.4 Exploratory biomarkers

Exploratory biomarkers of psychotic risk and potential functional measures of brain plasticity (Table 2) are being assessed to explore their association with the clinical response. Of note, is the novel automated speech analysis that is being utilized to generate acoustic parameters, which may serve as potential biomarkers to predict the onset of psychosis. During automated speech analysis, patients participate in audio‐recorded interviews to discuss dream reports and short‐term affective memories. Interview transcripts are subsequently analysed to extract acoustic parameters and generate word‐trajectory graphs and semantic features. Classifiers are applied to each to find the optimal combination to predict psychosis.

### 2.5 Substudies

Some patients are enrolling in an EEG and optional ocular safety substudy. Further details are described in the Appendix.

### 2.6 Statistical analyses

The sample size of 300 patients (150 per group) will provide 89% power to detect a 42.5% greater chance of achieving remission in patients with APS treated with BI 409306 versus placebo (given hypothesized remission rates of 57% [BI 409306] and 40% [placebo] at Week 52, with a dropout rate of 20%). A total of 133 events (remissions) will need to be observed to achieve full power. One formal, unblinded interim analysis will be conducted by an independent DMC when 67% of events have occurred, to consider stopping the trial for overwhelming efficacy if efficacy results cross a user‐defined alpha spending boundary with two‐sided alpha = 0.01 (criteria for stopping the trial at interim for overwhelming efficacy). The total alpha spent for the final analysis and outcome of the trial was 0.1 (10%). As the trial is of fixed duration, a blinded sample size reassessment, with the option to adjust the sample size, will be performed by the Trial Statistician when 90% of patients have been randomized or at the same time as the formal interim analysis (whichever occurs first).

Remission rates and time to remission, defined as start of treatment to remission and maintained until the end of the trial, will be analysed using the Wald test for treatment effect in a stratified Cox proportional hazards model at the two‐sided 10% significance level, stratified by baseline use of antipsychotics, and including and NAPLS risk score (Cannon et al., 2016) as a covariate and treatment effect as an independent variable. This model estimates the hazard ratio of BI 409306 versus placebo and the asymptotic 90% Wald confidence interval. Breslow's method for handling ties will be used. In the overall context of a Phase II setting, a 10% significance level was considered acceptable as a basis for deciding to continue with the trial. Secondary and further endpoints that measure time to an event will be analysed as per the primary endpoint. All change from baseline endpoints will be analysed using the restricted maximum likelihood based mixed‐effects model for repeated measures. Safety analyses will be descriptive, and the DMC will periodically review patient data to ensure patient safety. If there is a positive treatment response to BI 409306, pharmacogenetic analyses of genes associated with schizophrenia and NMDA receptor signalling will be conducted.

## 3 DISCUSSION

Individuals with APS have been identified by the American Psychiatric Association as a vulnerable group suffering from “manifest pathology and impaired function and distress” (American Psychiatric Association, 2013) and stand to benefit from early detection and treatment to alleviate symptoms and prevent possible psychotic conversion. However, current evidence‐based treatment options for patients with APS are limited. This is reflected in clinical guidelines, which provide mixed recommendations on the pharmacological and psychological interventions available in APS (American Psychiatric Association, 2013; European Medicines Agency, 2012; National Institute for Health and Care Excellence (NICE), 2013, 2014; Orygen: The National Centre of Excellence in Youth Mental Health, 2016; Schmidt et al., 2015). Sadly, a systematic review and meta‐analysis has demonstrated that current treatment options do not significantly reduce symptoms in patients at clinical high‐risk for psychosis (Devoe et al., 2018). However, another study has shown some benefit with cognitive behavioural therapy (CBT), which was associated with reduced symptoms at 12 months and a reduced risk of transition to psychosis at 6, 12, and 18–24 months (Hutton & Taylor, 2014). CBT is also recommended by the EPA as the first‐choice therapy in adult clinical high‐risk patients, combined with low‐dose second‐generation antipsychotics to manage symptoms and for the prevention of FEP (Schmidt et al., 2015). Nonetheless, these treatment options do not significantly improve functional outcomes in clinical high‐risk patients versus control conditions (Schmidt et al., 2015). Similarly, the PRIME North America study (Hawkins et al., 2008; Miller, Zipursky, et al., 2003) demonstrated that olanzapine did not significantly alter the neuropsychological course in a prodromal sample of symptomatic, treatment‐seeking patients (Hawkins et al., 2008). Although other clinical trials have shown some significant symptomatic benefits with olanzapine or antidepressants in clinical high‐risk patients, (Cornblatt et al., 2007; McGlashan et al., 2006) the effects of these compounds on conversion rates were either not significant versus controls or potentially due to patients with false positive prodromes in the prodrome‐positive study groups (Cornblatt et al., 2007; McGlashan et al., 2006). In addition, clinical findings have shown that ω‐3 polyunsaturated fatty acids may significantly improve symptoms, as measured by the Positive and Negative Syndrome Scale (PANSS) scores, and reduce the likelihood of psychotic onset in high‐risk patients (Amminger et al., 2015), although the effect on symptoms, as measured by Scale for the Assessment of Negative Symptoms, functioning and psychotic conversion were not replicated in a larger‐scale follow‐up study (McGorry et al., 2017). Finally, a network meta‐analysis of 16 randomized trials of pharmacological or psychological treatments in 2035 clinical high‐risk patients found that no intervention had a significant effect on the risk of transition to psychosis (Davies et al., 2018). Overall, patients with APS may benefit from new treatment options developed to alleviate the cognitive, behavioural, and emotional symptoms associated with APS and prevent or delay the onset of FEP.

Based on this unmet medical need, this large‐scale trial is investigating a pharmaceutical compound as part of a drug development program seeking worldwide regulatory approval for the treatment of APS. The compound under investigation is a novel PDE9 inhibitor, BI 409306, which is well tolerated in both young, healthy volunteers and in patients with schizophrenia (Boland et al., 2017; Brown et al., 2015; Moschetti et al., 2016; Wunderlich et al., 2015). In previous studies, the most frequently reported drug‐related adverse events were visual side effects that occurred shortly after dosing, were mild and mostly resolved within <1 h. Adverse events were generally restricted to high dose levels and were reversible (Boland et al., 2017; Moschetti et al., 2016; Wunderlich et al., 2015). This trial will further evaluate the visual safety of BI 409306 (50 mg twice daily), which has a satisfactory safety and tolerability profile (Boland et al., 2017; Moschetti et al., 2016), and will provide therapeutic exposure and functional target engagement as determined previously in the cerebral spinal fluid of healthy volunteers (Boland et al., 2017).

This study includes patients who are not routinely included in drug development trials (16–17 years of age), as the majority of clinical high‐risk patients are usually 16–18 years of age based on meta‐analysis and state‐of‐the‐art review (Fusar‐Poli et al., 2012; Fusar‐Poli et al., 2013). The upper age limit of 30 years was arbitrarily chosen, but is consistent with the clinical observation that patients >30 years of age are less likely to be diagnosed with APS and convert to psychosis. In our present study, patients receiving antipsychotics at baseline are included as it may be considered unethical by some to refuse antipsychotic treatment to clinical high‐risk patients. It is understood that this may be a potential confounder. For example, the use of antipsychotics may mask symptoms, affect remission of APS and affect the chances of conversion to psychosis (Schmidt et al., 2015). For that reason, patients are stratified by baseline use of antipsychotics, and the introduction of antipsychotic treatment between screening and baseline visit is not allowed.

Given the varying conversion rates seen in previous studies in this population (Addington et al., 2011; Cannon et al., 2008; Ruhrmann et al., 2010), the trial originally focused on the prevention of conversion to psychosis as the primary endpoint, including patients with an increased risk for psychotic conversion based on the NAPLS algorithm (Cannon et al., 2016). However, too many patients qualifying for an APS diagnosis were deselected based on NAPLS scores not meeting a ≥0.2 threshold, indicative of a >35% chance of conversion to psychosis at 52 weeks (Cannon et al., 2016). Therefore, it was deemed that psychotic conversion may be rare (Cannon et al., 2016) and a narrow outcome focus. Consequently, the trial was reoriented towards remission of APS as the primary endpoint, which, from the patients' perspective, is the more relevant outcome.

While remission is a reported outcome in many areas of schizophrenia and major depressive disorders research, adopting a dichotomously defined state change (in remission or not) as a primary endpoint is not without limitation. Most experienced psychiatrists are aware of the limitations and operate under conditions of dissonance in which management decisions are made based on a personal model of illness that has evolved from their own clinical experience (Craddock & Owen, 2007). Here, we defined remission from APS as a score of <3 on all the P1–P5 Positive Symptom items of SOPS and maintained until the end of treatment (a minimum of 6 weeks). Furthermore, we also measured change from baseline to end of study on several indicators of efficacy such as symptoms, cognition and functioning as continuous measures to support a diagnosis of remission. Unfortunately, adopting the dichotomous approach does not account for patients who initially enter remission, relapse, and then achieve remission again.

The primary endpoint and broad choice of secondary endpoints will help identify if BI 409306 is associated with symptomatic relief in nonconverters with APS through effects on symptoms (SOPS, PANSS), functioning (Schizophrenia Cognition Rating Scale), and cognition (tablet‐based Brief Assessment of Cognition). The key secondary endpoint of time to FEP, was chosen to assess whether BI 409306 can prevent or delay conversion to psychosis in a population of clinical high‐risk patients, using SIPS criteria for conversion to psychosis (McGlashan et al., 2010). The trial will also show whether treatment with BI 409306 can impact upon the development of comorbid psychiatric disorders, including depression and anxiety (Calgary Depression Scale, EuroQol‐5Dimensions‐5Levels) as well as other psychotic and nonpsychotic disorders (SIPS and Mini‐International Neuropsychiatric Interview) in APS. As clinicians require an effective means of identifying clinical high‐risk patients so that early treatment is possible, this trial may provide psychiatry with new, noninvasive biomarkers, and/or corroborate previous results to facilitate the transfer of promising biomarkers (Bedi et al., 2015; Cannon et al., 2016; Cornblatt et al., 2015; Ruhrmann et al., 2010) into daily clinical practice.

## 4 CONCLUSIONS

This trial will determine whether early intervention with BI 409306 50 mg twice daily for 52 weeks, provides clinical benefits in patients with APS by providing symptomatic relief and preventing the onset of FEP. This trial may identify new biomarkers of psychotic risk, which may improve the identification, intervention, and prevention of psychosis.

## CONFLICTS OF INTEREST

The authors met the criteria for authorship as recommended by the International Committee of Medical Journal Editors. R. S. E. K. is a paid consultant to Boehringer Ingelheim and several other pharmaceutical companies, and is the owner of VeraSci, which received paid services for this trial. R. S. E. K. also receives royalties for the tablet‐based Brief Assessment of Cognition (BAC) and the Brief Assessment of Cognition in Schizophrenia (BACS) symbol coding, which is part of the MATRICS battery. S. W. W., T. D. C., S. R., D. H. M., and P. M. are paid consultants to Boehringer Ingelheim International GmbH. T. D. C. has received grants from the National Institutes of Health. S. R. has received grants from the German Research Foundation, the European Commission, the Federal Ministry of Education and Research, and the Federal Institute for Drugs and Medical Devices, and has received speaker's honorarium from Boehringer Ingelheim International GmbH. D. H. M. is also a paid consultant to Alkermes, and has received grants from the National Institute of Mental Health, and honorarium from Alkermes. H. R. and S. P. are employees of Boehringer Ingelheim Pharma GmbH & Co KG and Boehringer Ingelheim International GmbH, respectively; M. S., D. C., D. R., and K. D. are employees of Boehringer Ingelheim Pharmaceuticals, Inc. None of the authors received direct compensation related to the development of this manuscript.

## AUTHOR CONTRIBUTIONS

**Richard S. E. Keefe**, **Scott W. Woods**, **Tyrone D. Cannon**, **Stephan Ruhrmann**, **Daniel H. Mathalon**, Philip McGuire, **Holger Rosenbrock**, **Kristen Daniels**, **Daniel Cotton**, **Dooti Roy**, **Stephane Pollentier**, and **Michael Sand:** Contributed to the trial concept and design. **Tyrone D. Cannon**: Also assisted in power calculations and contributed to the use of risk calculator used to ascertain those at greatest risk of developing psychosis. **Daniel H. Mathalon**: Also contributed to the design of the EEG substudy. All authors were involved in the preparation and review of the manuscript and approved the final version to be submitted.

## Supporting information

**AppendixS1**: supporting information

## Data Availability

No datasets were generated/analyzed during protocol development.
