# Supplementary material for: A randomized Phase II trial evaluating efficacy, safety, and tolerability of oral BI 409306 in attenuated psychosis syndrome: Design and rationale
Source: Early Interv Psychiatry. 2020 Dec 22;15(5):1315–25. doi: 10.1111/eip.13083 (PMC8451588; doi:10.1111/eip.13083)
Supplement: Supplementary file 1 — AppendixS1: supporting information [file EIP-15-1315-s001.docx]

# APPENDIX

# METHODS

## Optional EEG sub-study

Up to 80 patients with APS are taking part in an exploratory electroencephalogram (EEG) sub-study to assess the pharmacodynamic effects of BI 409306 on the central nervous system and assess if there is predictive validity for EEG and the onset of psychosis. The endpoint is neurophysiological change from baseline in quantitative EEG (qEEG), peak amplitudes (and latencies) of mismatch negativity (MMN), auditory and visual target P3b and novelty P3a, auditory N1 and visual P1, auditory steady-state response (ASSR) total power and inter-trial phase coherence for 20, 30, and 40 Hz driving conditions, and baseline (pre-stimulus) gamma power assessed during the ASSR paradigm. Assessments will be conducted at baseline and Week 52.

## Optional ocular safety sub-study

A sub-set of 50 patients will be enrolled from US sites to complete ocular safety assessments. Endpoints will include change from baseline in Best Corrected Visual Acuity test determined using a Snellen Chart and change from baseline in total error score from the Farnsworth-Munsel 100 hue testing.

# Table A1. Inclusion and exclusion criteria for the trial.

| Inclusion criteria | Exclusion criteria |
| --- | --- |
| - Meet the following diagnostic criteria for APS as defined by DSM-5(American Psychiatric Association, 2013) and determined by SIPS(McGlashan et al., 2010) at screening:   A) At least one of the symptoms of delusion, hallucinations, or disorganized speech in attenuated form, with relatively intact reality testing and is of sufficient severity or frequency to warrant clinical attention  B) Symptom(s) must have been present at least once per week in the last 1 month  C) Symptom(s) must have begun or worsened in the past year  D) Symptom(s) is sufficiently distressing and disabling to the individual to warrant clinical attention  E) Symptom(s) is not better explained by another mental disorder, including: Depressive or bipolar disorder with psychotic features and is not attributable to physiological effects of a substance or another medical condition  F) Criteria for any psychotic disorder have never been met   - SIPS interviews are video recorded and reviewed to confirm APS diagnosis - Age ≥16 and ≤30 years at the time of consent/assent - Male or female patients willing to use highly effective methods of contraception as per ICH M3 (R2)(International Conference on Harmonisation of Technical Requirements for Registration of Pharmaceuticals for Human Use M3 (R2), 2008) - Signed and dated written informed consent in accordance with GCP and local legislation prior to any trial-related procedures OR signed and dated informed consent provided by the patient’s parent(s) (or legal guardian) and assent by the patient prior to any trial-related procedures in accordance with GCP and local legislation. If the patient has a legal representative, then this legal representative must give written informed consent as well | - Present or past diagnosis of schizophrenia, schizophreniform, schizoaffective disorder, bipolar disorder I, or major depressive disorder with psychotic symptoms according to DSM-5 - Patients taking antipsychotic medication for less than 8 weeks or patients taking antipsychotic medication for a longer duration but who have not been on a stable dose for 8 weeks prior to informed consent - Patients who begin taking an antipsychotic between Visit 1 and Visit 2 - Patients who have discontinued antipsychotic medication less than 2 weeks prior to randomization - Patients taking clozapine - Any suicidal behavior in the past 2 years in the C-SSRS with a lethality of attempt ≥1, or with a lethality of 0 but a potential lethality of 2, or that in the judgment of the investigator would jeopardize the patient’s safety while participating in the trial - Any suicidal ideation of type 4 or 5 in the C-SSRS in the past 3 months (i.e. active suicidal thought with intent but without specific plan, or active suicidal thought with plan and intent) - In the judgment of the investigator, any clinically significant finding from the physical examination or laboratory value deviating from normal or any evidence of a clinically significant concomitant disease or any other clinical condition that would jeopardize a patient’s safety while participating in the clinical trial - Known diseases of the central nervous system (including but not limited to any kind of seizures or stroke) - History of significant head injury (>5 minutes without consciousness) - A serious developmental disorder that in the judgment of the investigator would inhibit the patient’s ability to comply with all trial procedures, or mental retardation (documented IQ <70), or acute attenuated symptoms exclusively related to intoxication from a psychotropic substance - Any documented active or suspected malignancy or history of malignancy within 5 years prior to screening, except appropriately treated basal cell carcinoma of the skin or in situ carcinoma of uterine cervix - Planned elective surgery requiring general anesthesia, or hospitalization for more than 1 day during the trial period - Significant history of drug or alcohol dependence or abuse (substance use disorder as defined in DSM-5) within the last 6 months prior to informed consent/assent - Patients who must or wish to continue the intake of restricted medications or any drug considered likely to interfere with the safe conduct of the trial - Patients taking strong or moderate CYP1A2 inhibitors who are also a CYP2C19 PM. Patients taking medication known to be a strong or moderate inhibitor of CYP1A2 must be prospectively genotyped to ensure they are not poor metabolizers of CYP2C19 - Patients taking strong or moderate CYP1A2 inhibitors who are also taking concomitant strong or moderate CYP2C19 inhibitors - Patients with a history of moderate to severe hepatic impairment (Child-Pugh B/C) - Patients with a history of moderate to severe renal impairment (Stage 3–5) - Women who are pregnant, nursing, or who plan to become pregnant while in the trial - In the judgment of the investigator, inability of the patient to comply with the clinical trial procedures - Currently enrolled in another investigational device or drug trial, or less than 30 days since ending another investigational device or drug trial(s), or receiving other investigational treatment(s) - Previous participation in any BI 409306 trial - Known hypersensitivity to the drug product excipients |

APS, attenuated psychosis syndrome; C-SSRS, Columbia Suicide Severity Rating Scale; CYP, Cytochrome P450; DSM-5, Diagnostic and Statistical Manual of Mental Disorders 5; GCP, Good Clinical Practice; ICH, International Conference on Harmonization; IQ, intelligence quotient; PM, poor metabolizers; SIPS, Structured Interview for Psychosis-Risk Syndromes.
